# Pre-clinical antigenicity studies of an innovative multivalent vaccine for human visceral leishmaniasis

**DOI:** 10.1371/journal.pntd.0005951

**Published:** 2017-11-27

**Authors:** Pedro Cecílio, Begoña Pérez-Cabezas, Laura Fernández, Javier Moreno, Eugenia Carrillo, José M. Requena, Epifanio Fichera, Steven G. Reed, Rhea N. Coler, Shaden Kamhawi, Fabiano Oliveira, Jesus G. Valenzuela, Luigi Gradoni, Reinhard Glueck, Gaurav Gupta, Anabela Cordeiro-da-Silva

**Affiliations:** 1 Parasite Disease group, Instituto de Investigação e Inovação em Saúde (i3S), Universidade do Porto, Porto, Portugal; 2 IBMC—Instituto de Biologia Celular e Molecular, Universidade do Porto, Porto, Portugal; 3 Departamento de Ciências Biológicas, Faculdade de Farmácia da Universidade do Porto, Porto, Portugal; 4 WHO Collaborating Centre for Leishmaniasis, Centro Nacional de Microbiología, Instituto de Salud Carlos III, Madrid, Spain; 5 Centro de Biología Molecular Severo Ochoa (CSIC-UAM), Universidad Autónoma de Madrid, Madrid, Spain; 6 Etna Biotech S.R.L, via Vincenzo Lancia, 57—Zona Industriale Blocco Palma 1, Catania, Italy; 7 Infectious Disease Research Institute (IDRI), Seattle, WA, United States of America; 8 Vector Molecular Biology Section, Laboratory of Malaria and Vector Research, NIAID, NIH, Rockville, MD, United States of America; 9 Unit of Vector-borne Diseases and International Health, Istituto Superiore di Sanità, Rome, Italy; Institut Pasteur de Tunis, TUNISIA

## Abstract

The notion that previous infection by *Leishmania* spp. in endemic areas leads to robust anti-*Leishmania* immunity, supports vaccination as a potentially effective approach to prevent disease development. Nevertheless, to date there is no vaccine available for human leishmaniasis. We optimized and assessed *in vivo* the safety and immunogenicity of an innovative vaccine candidate against human visceral leishmaniasis (VL), consisting of Virus-Like Particles (VLP) loaded with three different recombinant proteins (LJL143 from *Lutzomyia longipalpis* saliva as the vector-derived (VD) component, and KMP11 and LeishF3+, as parasite-derived (PD) antigens) and adjuvanted with GLA-SE, a TLR4 agonist. No apparent adverse reactions were observed during the experimental time-frame, which together with the normal hematological parameters detected seems to point to the safety of the formulation. Furthermore, measurements of antigen-specific cellular and humoral responses, generally higher in immunized *versus* control groups, confirmed the immunogenicity of the vaccine formulation. Interestingly, the immune responses against the VD protein were reproducibly more robust than those elicited against leishmanial antigens, and were apparently not caused by immunodominance of the VD antigen. Remarkably, priming with the VD protein alone and boosting with the complete vaccine candidate contributed towards an increase of the immune responses to the PD antigens, assessed in the form of increased *ex vivo* CD4^+^ and CD8^+^ T cell proliferation against both the PD antigens and total *Leishmania* antigen (TLA). Overall, our immunogenicity data indicate that this innovative vaccine formulation represents a promising anti-*Leishmania* vaccine whose efficacy deserves to be tested in the context of the “natural infection”.

## Author summary

Although vaccination is accepted as a potentially effective approach to prevent leishmaniasis, to date there is no vaccine available for human disease. The research on the topic is therefore extremely important, and the design and testing of new vaccine approaches, as well as non-traditional immunization schemes continues to be as relevant as before. This study proposes an innovative vaccine approach for human visceral leishmaniasis, not only due to its multi-antigen nature which contemplates both parasite and vector derived proteins, but also because it explores the possibility of the use of *Influenza* virosomes as antigen-delivery vehicles. A strong TLR-4 agonist completes the vaccine formulation. Here we show the rationale-behind this vaccine approach, the safety of all the vaccine components in our *in vivo* context, and immunogenicity studies of the optimized vaccine candidate in mice that explored the contribution of the virosome to the antigen-elicited immune responses. Additionally, we tested an unusual immunization scheme that potentiated the final vaccine-elicited immune responses. This prime-boost immunization approach gives relevance to the use of both parasite and vector derived antigens together as an anti-*Leishmania* vaccine, and proposes a new strategy for vaccination in endemic areas, where people are constantly exposed to sand fly bites.

## Introduction

Leishmaniasis is a spectrum of pathological outcomes caused by different *Leishmania spp*., intracellular parasites with a complex life cycle requiring a susceptible host and a permissive vector [1]. Visceral leishmaniasis, the most severe form of the disease, fatal if untreated, is caused by *L*. *donovani* and *L*. *infantum*, parasite species that migrate to the liver, spleen and bone marrow [2–4]. It has a worldwide distribution, being endemic in 74 countries, representing more than 37% of the total Earth terrestrial area [5]. Every year an estimated 0.2 to 0.4 million new VL cases occur and more than 20 000 people die, mostly in developing nations where access to healthcare is limited [6]. Furthermore, scarce and sometimes ineffective treatment options challenge leishmaniasis control [7].

Vaccination is considered one of the most cost/effective ways to control *Leishmania* infection. However, no human leishmaniasis vaccine is currently available. Several candidates have been proposed during the past few decades [8]. Some were shown to be immunogenic and have conferred protection against *Leishmania* in rodent models. Nevertheless, most of them were discarded after proving to be ineffective in large animals [8, 9]. Furthermore, most of these studies shared a limitation which may have been responsible for the overestimation of the vaccine candidates effectiveness: they had a binomial focus (host-parasite) and disregarded the contribution of the vector, essential in vaccine efficacy determination as highlighted by Peters *et al* who showed the loss of protection of a potentially-good vaccine candidate when tested in the context of vector-transmitted leishmaniasis [10].

*Leishmania* parasites are transmitted by sand flies from the genera *Lutzomyia* and *Phlebotomus* in a specific vector-*Leishmania spp*. pairing [11]. During the sand fly blood meal, parasites together with vector derived factors, including saliva, are introduced into host skin [11–13]. Previous exposure to sand fly salivary components has been shown to confer protection against vector-transmitted *Leishmania* [14, 15]. Furthermore, in recent studies, protection against natural transmission of *Leishmania* has been attained by vaccination with defined salivary molecules in animal models for both cutaneous leishmaniasis and VL [16, 17]. Interestingly, these proteins were shown to improve the protection induced by live anti-*Leishmania* vaccines [18, 19]. Fundamentally, the Th1 immune response elicited against a salivary molecule can adversely impact parasite establishment in the host.

This study proposes a novel vaccine candidate based on defined antigens of both parasite (KMP11 and LeishF3+, the latter a fusion protein consisting of Nucleoside hydrolase, Sterol 24-c-methyltransferase and Cysteine protease B), and sand fly vector (salivary protein LJL143) origins, formulated into *Influenza* virosomes and adjuvanted with GLA-SE, a TLR-4 agonist. The sand fly antigen LJL143 was shown to produce a long lasting Th1 immune response in dogs, which impacted parasite growth *in vitro* [20]. One of the parasite-derived antigens, KMP-11, was already demonstrated to be individually effective against VL in the pre-clinical context [21], as were each of the individual components of the second parasite-derived antigen, the fusion protein LeishF3+ [22–24]. Additionally, LeishF3+ predecessor antigen (a fusion protein consisting of Nucleoside hydrolase and Sterol 24-c-methyltransferase, but not Cysteine protease B), was considered safe and immunogenic in the clinical context (Phase I trial) [25], as were also both the adjuvant and the virosomes [26, 27]. *Influenza* virosomes represent a unique vaccine delivery system, flexible but robust, that allows loading of a wide variety of antigens [28, 29]. The VLP-based antigen formulation has the potential to generate both CD4^+^ and CD8^+^ specific memory T cells, the latter due to the potentiation of cross-presentation events [30]. The immune response elicited by the immunization should induce a Th1 phenotype due to the adjuvant chosen and the presence of the sand fly salivary antigen [26, 31]. In theory, an immunized individual bitten by an infected sand fly and exposed to parasites and vector saliva, will quickly mount both a strong Th1 anti-*Leishmania*, and a strong Th1-DTH anti-sand fly saliva immune responses, resulting in prevention of infection establishment.

Here, we explore the safety and antigenicity of the vaccine candidate, using *ex-vivo* and *in-vivo* approaches.

## Materials and methods

### Ethics statement

Animal experiments were performed in accordance with the IBMC.INEB Animal Ethics Committee and the Portuguese National Authorities for Animal Health guidelines (directive 2010/63/EU). BPC and ACdS are accredited for animal research (Portuguese Veterinary Direction—DGAV, Ministerial Directive 113/2013). DGAV approved the animal experimentation presented in this manuscript under the license number 0421/000/000/2013.

The study with human Peripheral Blood Mononuclear Cells (PBMCs) was approved by the Hospital de Fuenlabrada (Madrid, Spain) Ethics and Research Committee (protocols APR12-65 and APR14-64), and all participants gave written informed consent to be involved.

### Antigens and Adjuvant

#### KMP11

The *L*. *infantum* gene coding for KMP11 (LinJ.35.2260) was cloned in the vector pET-28b for expression in *E*. *coli* BL21 (DE3). Briefly, the gene was amplified from *Leishmania* genomic DNA (strain JPC) using the oligonucleotides 5’-CCATGGCCAC CACGTACGAG G (Fw; underlined is the *Nco*I restriction site) and 5’-GGATCCTTAC TTGGACGGGT ACTGCG (Rv; underlined is the *Bam*HI restriction site), and subcloned into pET-28b using a NcoI/BamHI restriction approach. The final construct (pET-LiKMP11; confirmed by DNA sequencing) was then transformed into *E*. *coli* BL21 (DE3) by electroporation. Protein expression was achieved by aired incubation of a bacterial suspension (OD600 0.6–0.8) for 4 hours at 37°C, under selective pressure (25 μg/ml of kanamycin) and IPTG induction (1 mM). Protein was purified first by stepwise ammonium sulfate precipitation (KMP11 did not precipitate until protein solution arose an 80% ammonium sulfate saturation) and afterwards by anion-exchange chromatography (DEAE-Sephacel; KMP11 eluted in 10 mM Tris/HCl (pH = 8,5), 150 mM NaCl). Finally, the protein was passed through a polymyxin-B agarose matrix (Sigma-Aldrich, MO, USA) for endotoxin removal. The purity was higher than 95%, as determined by SDS-PAGE and Coomassie staining.

#### LeishF3 and LeishF3+

The fusion protein LeishF3, used only during the optimization process of the vaccine candidate, was produced and purified as described elsewhere [25]. The upgraded version of LeishF3, LeishF3+ was originated by the fusion of an additional *Leishmania* antigen (CPB) to the two that constitute LeishF3 (NH and SMT), in a way to increase epitope diversity and consequently enhance human T cell recognition. Production and purification processes used are similar to the ones used previously [25].

#### LJL143

The sandfly salivary protein used during the optimization process of the vaccine candidate (his-tagged) was produced using a mammalian expression system, as explained elsewhere [20]. LJL143 used in the antigenicity pre-clinical assay *per-se* (non his-tagged) was obtained using a yeast expression system (more cost-effective). Briefly, DNA coding for LJL143 without the signal peptide was codon optimized based on *Pichia pastoris* usage preference and subcloned into Pichia secretory expression vector pPICZαA (Invitrogen) using EcoRI/XbaI restriction sites. The correct insert sequence and reading frame of recombinant plasmid was confirmed by double-stranded sequencing using vector flanking primers α-factor and 3’AOX-1 and then transformed into *Pichia pastoris* X-33 by electroporation. The expression of LJL143 was induced with 0.5% methanol at 30°C for 72 hours and the highest expression clone was chosen for making seed stock with 20% glycerol. Large-scale expression of LJL143 was induced with methanol in 10L fermentation. Coomassie G-250 (Simply Blue) stained NuPAGE Bis-Tris gels (Invitrogen) were used to assess the purity of the recombinant protein.

#### Adjuvant

The synthetic TLR-4 agonist Glucopyranosyl Lipid A (GLA-SE) was produced and provided by IDRI, as previously reported [32].

### VLP-based antigen formulation process

Four different virosomal preparations have been specifically designed for this study, three of them containing each of the individual antigens, and one containing all the three antigens together. Briefly, a solution containing 1 mg of inactivated *Influenza* virus A/H1N1/California was pelleted at 286 000g for 1 hour, dissolved in presence of 0.5 ml of PBS containing 0.1 M of Octaethyleneglycol mono (n-dodecyl) ether (OEG; Sigma Aldrich, MO, USA), and then mixed with 32 mg of phosphatidylcholine (Lipoid Ag, Steinhausen, Switzerland) dissolved in 1.5 ml of PBS-OEG 0.1 M. The mixture was centrifuged at 100 000g for 30 min and the supernatant containing Haemagglutinin and Neuraminidase was recovered. For the individual virosomal formulations, the obtained supernatant was then mixed with 2 mg of Leish-F3 (or Leish-F3+), or KMP11 or LJL143 in presence of detergent. Virosomes were then formed by detergent removal and sterile-filtered. The virosome particles containing the mixture of the three antigens were produced similarly, from 1 mg of starting influenza protein mixed with 1 mg of each antigen (Leish F3+, KMP11 and LJL143). Size determination and distribution of the particle population was performed using a Zetasizer Nano instrument (Malvern Instruments, Malvern, UK). Parasite derived and/or VD proteins content in virosome particle was determined by SDS-PAGE Coomassie Stained.

### Human PBMCs *ex vivo* assays

Subjects included in this study were residents of a *L*. *infantum* post-outbreak area. Up to 14 healthy endemic individuals (theoretically never exposed to *Leishmania*), 11 asymptomatic subjects (positivity to the *in vitro* PBMC proliferation assay to soluble *Leishmania* antigen) and 21 cured VL patients (clinically diagnosed with VL; presence of *Leishmania* confirmed in blood by PCR; three months after successful treatment with liposomal amphotericin B) were included in the antigenicity assays. Blood samples were collected at the hospital blood bank and the internal medicine department (Hospital of Fuenlabrada, Madrid). PBMCs were prepared by density gradient centrifugation of heparinized blood samples (Lymphocyte Isolation Solution, RAFER, Spain). PBMCs were adjusted up to 2×10^6^ cells/ml in complete medium (RPMI 1640 supplemented with 100 U/ml penicillin, 100 μg/ml streptomycin, 2mM L-glutamine, 25mM HEPES and 10% heat inactivated fetal calf serum), and cultured in 96-well plates at a density of 2×10^5^ cells per well for 5 days with either KMP11 (10 μg/ml), LeishF3 (10 μg/ml), LeishF3+ (10 μg/ml), LJL143 (10 μg/ml), soluble leishmanial antigen—SLA (10 μg/ml) or PHA-M (5 μg/ml) in a final volume of 200 μl per well. The supernatants of the *in vitro* cell cultures were collected and stored at -20°C for cytokine quantification. Interferon-γ, granzyme B, TNF-α, and IL-10, were quantified in culture supernatants, using the BD Cytometric Bead Array Human Flex Set (BD Biosciences, NJ, USA) following the manufacturer’s instructions. Data were acquired using a FACSCalibur flow cytometer and analyzed using the Flow Cytometric Analysis Program Array (BD Biosciences, NJ, USA).

### Mice

Six to eight weeks old male BALB/c mice (Charles River Laboratories, France) were maintained under specific-pathogen free conditions at the IBMC facilities, with water and food *ad libitum*.

### General *in vivo* experimental layout

Animals were immunized intramuscularly with a maximum of 50 μl of the respective formulation in the thigh. The volumes administered were based on the concentration of the antigen/adjuvant preparations, and adjusted to equivalent final volumes with PBS. Unless otherwise stated, BALB/c mice were immunized three times at four weeks intervals, in the two thighs alternately. Four weeks after the last immunization, animals were euthanized by cervical dislocation, under volatile anesthesia (Isoflurane, Piramal healthcare, Northumberland, UK).

### Mice immunizations

Two major *in vivo* experimental set ups originated this work, the first one for optimization purposes, and the second one as the actual pre-clinical trial.

#### Optimization of the vaccine candidate: Determination of the best antigen and adjuvant doses

Seven BALB/c mice/group were immunized with 1 μg or 5 μg of adjuvant and 1 μg or 5 μg of each individual virosomal preparations of LJL143, KMP11 and LeishF3 (formulated-antigens, VPA). In parallel, fourteen BALB/c mice received adjuvanted non-formulated antigens (PA) in the same two doses (1 and 5 μg). One μg of non-adjuvanted antigens (P) or PBS were injected in control animals.

#### Optimized-vaccine pre-clinical trials

Seven BALB/c mice/group were immunized with adjuvanted non-formulated proteins [1 μg of each component; PA (1+1+1)] or with adjuvanted formulated antigens (VPA). Two different VPA combinations were tested regarding antigen quantities: 1+1+1 or 1+5+5 indicate the administered dosages in μg of LJL143, KMP11 and LeishF3+ (individual virosome formulations). VPA (Mix) refers to the third virosome formulation tested, in which three antigens (1 μg, each) were simultaneously formulated in the same virosome. In parallel, an extra group received adjuvanted non-formulated proteins (GLA-SE+KMP11+LJL143+LeishF3+; 1 μg each), three weeks after priming with 1 μg of non-adjuvanted LJL143 (Pre-LJL PA). Only the adjuvant (A) or the adjuvanted empty-virosome (VA) were injected in the negative control groups. The experimental timeline is represented in S2 Fig.

### Mice whole blood/sera collection

Blood from mice was collected through intracardiac puncture under isoflurane anesthesia. One hundred μl of blood were immediately dispensed to a pre-heparinized tube to be used for general hematological determinations. The remaining volume was left to clot, and serum was then collected and stored at -80°C for posterior immunoglobulin titration.

### Splenic aseptic collection and processing

Mice were disinfected using 70% ethanol. Thereafter, abdominal skin was cut with sterile scissors and removed to expose the abdomen. Peritonea were then opened using a new pair of sterile scissors and tweezers, and the spleens harvested to pre-weighed 15 mL falcon tubes containing 5 ml of complete RPMI (Lonza, Switzerland) [10% heat-inactivated FBS (Lonza, Switzerland), 2 mM L-glutamine, 100 U/ml penicillin and 100 μg/ml streptomycin (BioWhittaker, Walkersville, MD, USA)] supplemented with 50 μM 2-mercaptoethanol (Sigma-Aldrich, MO, USA). Falcon tubes were re-weighed to obtain spleen masses. Splenic single cell suspensions were then obtained using Falcon^TM^ Cell Strainers (Fisher Scientific, MA, USA), and their concentrations determined using an EVE^TM^ automatic cell counter (NanoEntek, Seoul, Korea).

### Cellular proliferation assays

Ten million cells per animal were pelleted, washed twice with PBS and stained for 10 minutes at 37°C with CFSE (1 μM; in 1 ml of PBS; Thermo Fisher Scientific, MA, USA). Complete RPMI was then added to cell suspensions to stop the reaction, that were then pelleted, re-suspended in complete RPMI and incubated at 4°C for 5 minutes. Afterwards, cells were once more centrifuged (5 min, 350 g), resuspended in complete RPMI supplemented with 50 μM 2-mercaptoethanol (Sigma-Aldrich, MO, USA), and plated into u-bottom 96-well plates at the final amount of 2.5 x 10^5^ cells per well. After plating, different stimuli were added, depending on the experiment: individual non-formulated antigens (KMP11, LJL143 or LeishF3(+); 10 μg/ml), a pool of the three recombinant antigens (KMP11+LJL143+LeishF3+; 10 μg/ml each), and total *Leishmania* antigen (TLA; equivalent to 10 parasites per cell). Concanavalin A (3 μg/ml) and complete RPMI medium were added as positive and negative controls, respectively. From each animal, a single CFSE staining was performed, being only then the cells divided and stimulated. This warrants that any proliferating cells, regardless the condition, comes from the same initial suspension. Cells were incubated (37°C and 5% CO_2_) for three (positive controls) or four days (remaining stimuli), and then the originated cell culture supernatants were collected and stored at -80°C for cytokine quantification. Each determination was performed in duplicate. Proliferating CD4^+^ and CD8^+^ cell populations were determined by Flow Cytometry, based on the premise that CFSE intensity gradually decreases after each cell division.

### Flow cytometry

The anti-mouse monoclonal antibodies used to perform this study were all purchased from BioLegend (CA, USA) unless otherwise stated: FITC labeled anti-MHC-II(I-Ad) (AMS-32.1, BD Biosciences, NJ, USA), anti-IFN-γ (XMG1.2) and anti-IL-17A (TC11-18H10.1); PE labeled anti-CD8 (53–6.7, BD), anti-Siglec-F (E50-2440, BD), anti-IL-4 (11B11) and anti-IL-6 (MP5-20F3); PerCP-Cy5.5 labeled anti-Ly6C (HK1.4) and anti-TNFα (MP6-XT22); PE-Cy7 labeled anti-CD3 (HA2) and anti-CD11b (M1/70); APC-Cy7 labeled anti-CD11c (N418); APC labeled anti-CD19 (6D5), anti-IL-5 (TRFK5) and anti-IL-10 (JES5-16E3); BV510 labeled anti-CD4 (RM4-5) and Pacific Blue^TM^ labeled anti-Ly6G (1A8).

To analyze lymphoid and myeloid cell populations, two panels of antibodies were designed. The lymphoid panel was composed of anti-CD8, -CD3, -CD4, and -CD19. The Myeloid panel comprised anti-CD11b, -CD11c, -Siglec-F, -Ly6C, -Ly6G and -MHC-II. Surface staining of splenic cells was performed in PBS + 0.5% BSA (20 min, 4°C) followed by 15 min fixation with 2% PFA. For intracellular staining (non-specific cytokine production), splenocytes were cultured for 2h with PMA/Ionomycin (50/500 ng/ml) and then Brefeldin A (10μg/mL) was added for 2 additional hours. Cells were surface stained, fixed and permeabilized with 1% saponin (Sigma-Aldrich, MO, USA) and then intracellularly stained [33]. Samples were acquired in a FACSCanto (BD) and analyzed with FlowJo software v10 (TreeStar, OR, USA).

An initial gate plotting FSC-A versus SSC-A was performed. Afterwards, singlets were selected by plotting FSC-A versus FSC-H and the remaining cell populations were resolved. T lymphoid cell populations were defined as CD3^+^/CD4^+^ and CD3^+^/CD8^+^ while B cells were defined as CD19^+^. Non-specific cytokine production by T cells was assessed within CD3^+^/CD4^+^ and CD3^+^/CD8^+^ cells. Myeloid cell populations were gated as eosinophils (Siglec-F^+^/SSC-H^int/high^), neutrophils (CD11b^high^/Ly6G^high^/Siglec-F^-^), DCs (CD11c^+^/MHC-II^int/high^) and monocytes/macrophages (CD11b^+^/CD11c^-^/Ly6G^-^/Siglec-F^-^). Proliferating T cells (CD4^+^ or CD8^+^) were defined as CFSE^int/low/neg^ (FITC channel), always comparing each condition with the respective negative control.

### Determination of mouse cytokines by ELISA

Cytokines were quantified, according to the manufacturer, using the commercial kits: Mouse IL-10 DuoSet ELISA, (R&D Systems, MN, USA), IL-12p70, IL-4 and IFN-γ ELISA MAX Deluxe (BioLegend, CA, USA).

### Hematological parameters determination

Uncoagulated murine blood samples were used to obtain a complete blood evaluation, including haemoglobin and hematocrit levels, and total red blood cell, white blood cell, and platelet counts, using an automated blood cell counter (Sysmex K1000, Hamburg, Germany).

### Serum immunoglobulins titration by ELISA

For specific immunoglobulin titration assays, high protein binding 96-well plates were coated overnight at 4°C, individually with each one of the three antigens comprising the vaccine formulation (1 μg/ml), with a pool of the three antigens (1 μg/ml each), or with soluble *Leishmania* antigen (SLA; 1 μg/ml); all solutions were prepared in NaHCO3 0.1 M. Additionally, total IgG levels were also determined, using as a coating agent α-mouse IgG (1 μg/ml; Southern Biotech, AL, USA). Plates were then washed with PBS Tween 0.1%, blocked with 1% gelatin in PBS (blocking buffer) for 1 hour at 37°C and re-washed. Each serum was then serially diluted (twofold, 7 dilutions) in blocking buffer. Wells filled with just blocking buffer were used as blanks. Plates were incubated for 1 hour at 37°C and re-washed. Afterwards IgG and isotypes, IgM and IgE were detected using horseradish peroxidase (HRP) coupled α-mouse antibodies [diluted 1:5000 (IgM, IgE, IgG1, IgG3, IgG2b and IgG2a; Southern Biotech, AL, USA) or 1:8000 (IgG; Southern Biotech, AL, USA) in blocking buffer; incubated for 30 minutes, at 37°C]. The plates were washed for a last time, and the substrate (orthophenyldiamine (OPD) in citrate buffer) was added for 10 minutes, time after which the reaction was stopped with HCl 3 N. Absorbance values were determined at 492 nm in a Synergy^TM^ 2 Multi-Mode Reader (BioTek instruments, VT, USA).

The value of the last dilution factor for which the corrected optical density was equal or higher than 0.1 was the defined titer of the antibody (endpoint titer), as has been previously described [34].

### Statistical analysis

Results are generally expressed per individual animals/samples, with a representation of the group mean value ± standard deviation. Statistical differences were analyzed using GraphPad Prism v6.01 (CA, USA). Mice experimental groups were compared using either the one-way ANOVA or the unpaired t-test. Comparisons between human samples were performed using Mann–Whitney test.

## Results

### Rationale behind the vaccine formulation and optimization steps

#### Optimal antigen/adjuvant doses

**Fig 1:**
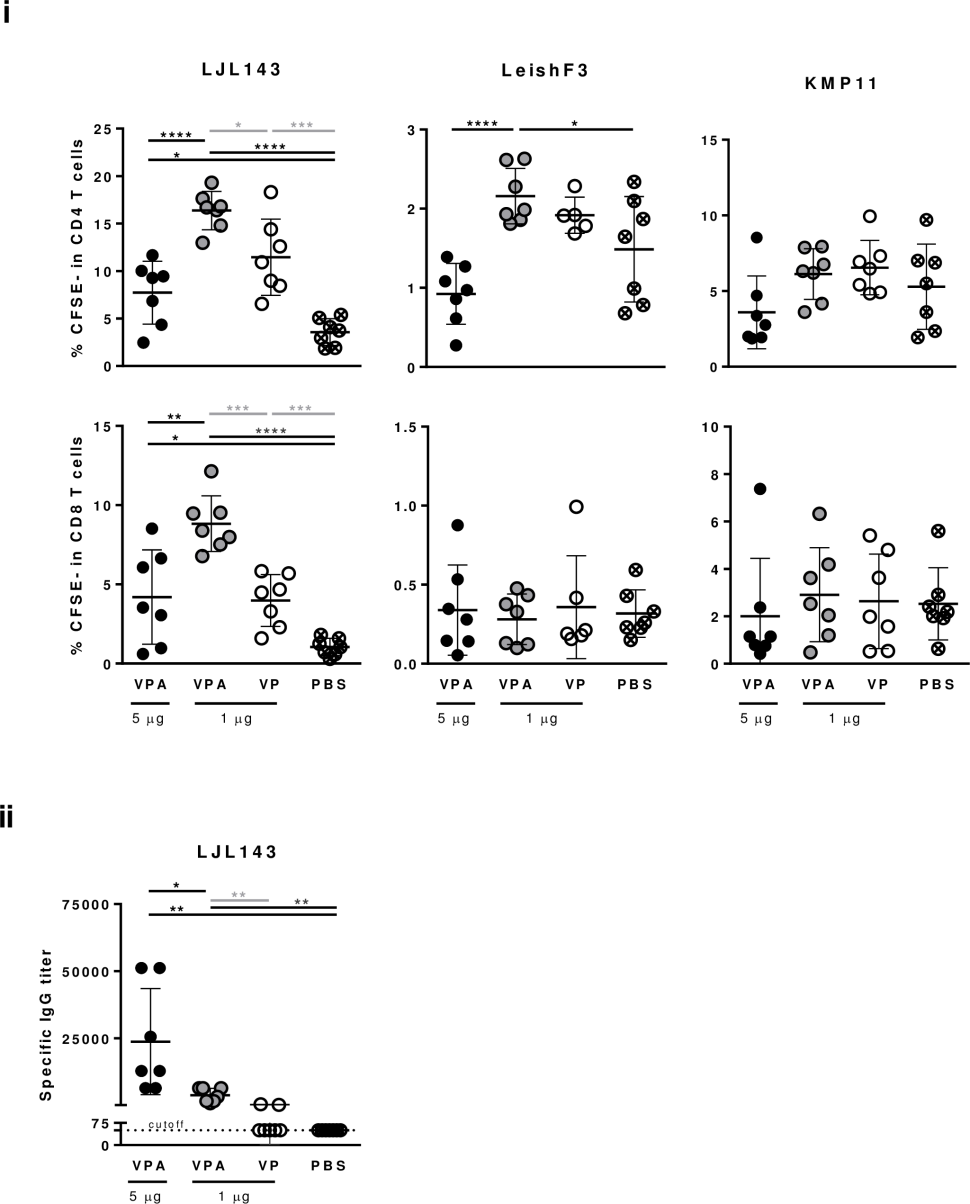
One microgram of adjuvant/formulated-antigens generates a higher specific cellular immune response than five micrograms. Groups of BALB/c mice were immunized 3 times i.m. (separated by 4 weeks each) with 2 different doses of adjuvant/formulated-antigens: 1 μg and 5 μg of each individual component (VPA). Non-adjuvanted formulated antigens (VP; 1 μg of each antigen) or PBS were injected in animals from the 2 control groups. Four weeks after the last immunization, animals were euthanized and their spleens and sera collected. (i) Specific CD4^+^ and CD8^+^ T cell proliferation was assessed by Flow Cytometry four days after CFSE-stained splenocytes-culture in the presence of LJL143, LeishF3 or KMP11 (10 μg/ml). (ii) Serum antigen specific IgG titers for LJL143 were determined by ELISA. Each dot represents one animal. Average and SD of the values within each group are shown. Statistical differences are properly identified (Unpaired t-test: * p≤0.05, ** p≤0.01, *** p≤0.001 and **** p≤0.0001).

To determine if the specific responses elicited by vaccination are dependent on the doses of antigens/adjuvants, two different antigen/adjuvant amounts were tested *in vivo*. Initially, vaccine-elicited specific humoral and cellular immune responses were evaluated in mice immunized with either 1 μg or 5 μg of adjuvant/formulated-antigens (VPA) and compared with those obtained for control groups that received either non-adjuvanted formulated antigens (VP; 1 μg of each antigen) or PBS. The specific CD4^+^ and CD8^+^ T cells proliferation levels determined against LJL143, as well as the IgG antibody titers detected were higher in animals immunized with the adjuvanted (VPA; 1 μg of antigen) in comparison with animals immunized with the non-adjuvanted (VP; 1 μg of antigen) formulations (Fig 1i and 1ii; p≤0.05, p≤0.001 and p≤0.01, respectively), indicating the necessity of the adjuvant inclusion in the vaccine formulation. Interestingly, the CD4^+^ proliferating T cell levels in response to both LJL143 and LeishF3 were higher in animals that received 1 μg of formulated-antigens/adjuvant in comparison with those that received 5 μg (Fig 1i; p≤0.0001). The same response was observed for CD8^+^ proliferating T cell levels against LJL143 (p≤0.01), while no differences between groups were detected in response to LeishF3 (Fig 1i). Of note, the responses against LeishF3 were weaker compared to those mounted against LJL143, and the magnitude of the immune response was higher for CD4^+^ than CD8^+^ T cells (Fig 1i). There was no difference in the proliferation of either CD4^+^ or CD8^+^ T cells between groups in response to KMP11 (Fig 1i). The picture concerning the elicited specific humoral immune response is reversed. The detected IgG titers specific for LJL143 were higher in animals that received 5 μg of formulated-antigens/adjuvant, compared with ones that received 1 μg (p≤0.05) (Fig 1ii). Humoral reactivity was not detected in this experiment against either LeishF3 or KMP11.

A similar experimental layout was used to assess responses in animals that received adjuvanted non-formulated antigens (PA) in the same two doses (1 and 5 μg), in comparison with control groups that received either non-adjuvanted antigens (P; 1 μg of each antigen) or PBS. An adjuvant effect on the antigen-elicited immune responses was observed for LJL143 and LeishF3 (S1i and S1ii Fig; PA *versus* P; p≤0.05–0,001). Furthermore, without formulation, LJL143 retained its higher immunogenicity compared to LeishF3 and KMP11 (S1i and S1ii Fig). Moreover, the two doses of antigens induced similar CD4^+^ and CD8^+^ T cell responses against LJL143; the responses were higher in animals that received 1 μg compared to 5 μg for LeishF3, and were similar to controls for KMP11 (S1i Fig). In line with the verified for formulated antigens, the detected specific humoral responses against both LJL143 and LeishF3 were higher in animals that received 5 μg of antigens/adjuvant and were not detected in any of the KMP11-immunized groups (S1ii Fig).

#### Immune recognition of the antigens by individuals from a VL-endemic area in Spain

**Fig 2:**
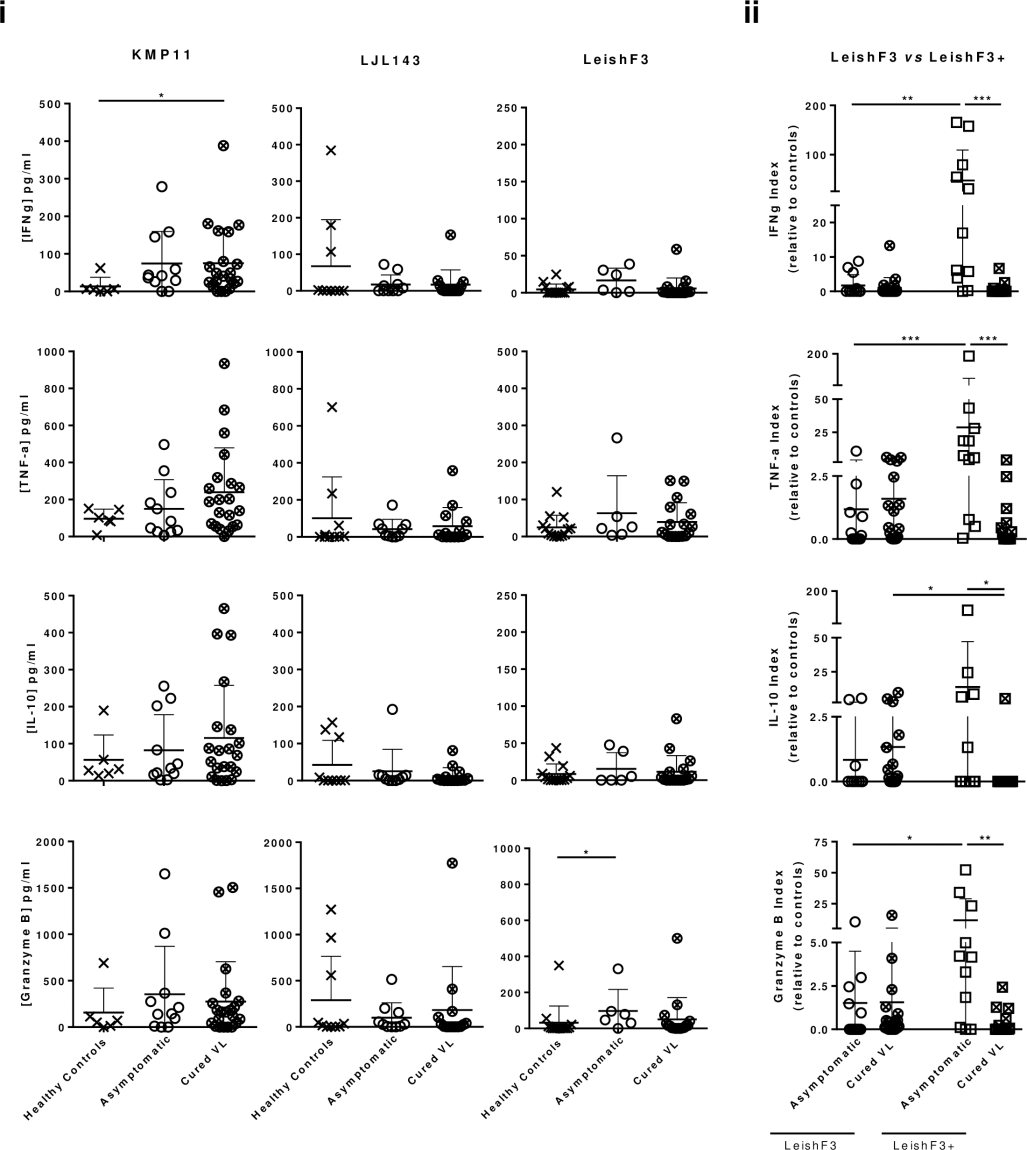
Antigen recognition in patients from a *L*. *infantum* endemic area in Spain. PBMCs were purified from peripheral blood collected from asymptomatic and cured VL patients or matched endemic controls. (i) Two hundred thousand cells were stimulated for 5 days with either KMP11 (10 μg/ml), LeishF3 (10 μg/ml) or LJL143 (10 μg/ml). Specific cell responses were identified through quantification of IFN-γ, TNF-α, IL-10 and Granzyme B in the resulting culture supernatants. (ii) A similar proliferation experiment was performed to compare human responses induced by the same amount (10 μg/ml) of LeishF3, and of its improved version LeishF3+; presented data resulted from the normalization of the determined cytokine absolute values, in relation to the average values determined for endemic controls. Each dot represents one individual. Average and SD of the values within each group are shown. Statistical differences are properly identified (Mann–Whitney test: * p≤0.05, ** p≤0.01 and *** p≤0.001).

The different immune response-magnitudes induced by the antigens in mice indicated that a final step to assess the immunogenicity of each antigen in humans, *ex-vivo*, was needed. Individual specific recognition of LJL143, LeishF3 and KMP11, was evaluated in PBMCs from asymptomatic and cured VL patients from a leishmaniasis high-incidence area (Madrid, Spain), by measuring the specific cytokine profile on supernatants after five days of stimulation. In parallel, PBMCs from endemic healthy individuals were used as controls. Remarkably, stimulation with KMP11 induced a better response. Samples from cured VL and asymptomatic patients, significantly (p≤0.05) and tendentiously secreted more IFN-γ than the controls (Fig 2i). Additionally, TNF-α and IL-10 responses generated against KPM11 were also tendentiously higher comparing infected (symptomatic and cured) with controls, while Granzyme B responses (excluding some outliers) were generally comparable among groups (Fig 2i). On the other hand, the responses detected against LeishF3 were lower in magnitude, and generally similar comparing the infected (asymptomatic and cured) with the controls (Fig 2i). Curiously, PBMCs from some individuals of the three different groups (Old World human samples) responded to LJL143, a salivary protein from *L*. *longipalpis*, the vector of VL in the New World (Fig 2i).

Due to the weak immunogenic response obtained against LeishF3 in humans, the immunogenicity of an improved version of this antigen, named LeishF3+ was assessed through the same *ex vivo* stimulation approach. Basically, to enhance human T cell recognition we increased epitope diversity by fusing an additional antigen, CPB, to LeishF3, to generate LeishF3+. As a mean of comparison, stimulation with LeishF3 and LeishF3+ was performed in parallel. Interestingly, the responses detected against LeishF3+, in terms of IFN-γ, TNF-α and Granzyme B were significantly improved, only in the group of samples from asymptomatics, in comparison with the ones induced by LeishF3 (Fig 2ii; p≤0.01, p≤0.001 and p≤0.05, respectively). On average, the IFN-γ, TNF-α, IL-10 and Granzyme B responses of samples from asymptomatic patients detected against LeishF3+ were 50, 25, 15 and 10 fold higher than the responses obtained for control samples (Fig 2ii).

### Vaccine pre-clinical antigenicity tests in mice

Different experimental groups were designed for the pre-clinical tests of the optimized vaccine candidate in mice to assess, besides the safety of the vaccine components, the influence of several variables in the final outcome of the immunization, such as the contribution of the virosome to the induction of immunogenicity or the possibility of immunodominance of the sand fly salivary protein (S2 Fig). In parallel, the effect of a prime with the sand fly-salivary protein in the final vaccine-elicited immune response was evaluated (S2 Fig). Defined above as essential to increase the antigens immunogenicity, the adjuvant (GLA-SE) was administered to all groups. Human immunogenicity studies dictated the replacement of LeishF3 by LeishF3+ as one of the three antigens of the optimized-vaccine.

#### Safety profile of the vaccine components

The safety profile of the different components was extrapolated by the determination of general hematological parameters in whole blood, antibody-specific IgE titers in serum, and cellular composition of the splenic cell compartment. The hematological parameters determined were comparable among groups and were within their normal ranges (Table 1). The spleen weights and cell numbers were also comparable among groups (S3i Fig). Consequently, the determined absolute numbers of lymphoid and myeloid splenic cell populations, were also in the magnitude of the normal ranges, and overall comparable among groups (S3ii and S3iii Fig). Furthermore, the responsiveness of CD4^+^ T cells, evaluated through cytokine production after non-specific stimulation, was similar among groups (polyfunctional CD4^+^ T cells; S3iii Fig), and no antigen-specific IgE titers were detected (S3iv Fig). Overall, the full panel of results points to the safety of all the vaccine components.

**Table 1:** Hematological profile determined in the optimized vaccine pre-clinical trials.

	Erythrocytes (x 10^12 cells/L)	Hemoglobin (g/dL)	Hematocrit (%)	Leukocytes (x 10^9 cells/L)	Platelets (x 10^9 cells/L)
**VPA (1+1+1)**	8.299 ± 0.5661	13.51 ± 0.7712	40.54 ± 2.989	5.129 ± 1.810	568.6 ± 113.1
**VPA (1+5+5)**	8.539 ± 0.6421	13.76 ± 0.8404	41.14 ± 3.392	4.714 ± 1.593	507.6 ± 97.93
**VPA (Mix)**	9.014 ± 0.1952	13.87 ± 0.4424	45.49 ± 1.463	5.514 ± 1.389	517.6 ± 59.46
**VA**	8.356 ± 0.4527	13.40 ± 0.5538	40.61 ± 2.389	5.929 ± 1.665	415.9 ± 144.9
**A**	8.917 ± 0.8441	13.89 ± 1.051	44.50 ± 4.941	6.586 ± 1.710	389.9 ± 55.54
**PA (1+1+1)**	8.713 ± 0.3155	13.51 ± 0.4706	43.63 ± 2.094	4.771 ± 1.627	510.4 ± 157.8
**Pre-LJL PA**	8.681 ± 0.5582	13.40 ± 0.7141	42.80 ± 3.180	7.8 ± 2.998	386.1 ± 131.5

The values shown represent the mean ± standard deviation. Reference intervals, according to Charles River Laboratories (8–10 week male mice) are: 6.93–12.24 x 10^12^ cells/L for erythrocytes, 12.6–20.5 g/dl for hemoglobin, 42.1–68.3% for hematocrit, 3.48–14.03 x 10^9^ cells/L for leukocytes and 420–1698 x 10^9^ cells/L for platelets.

#### Immunogenic profile of the optimized vaccine

To determine the immunogenicity of the optimized vaccine (normal immunization scheme), specific-elicited humoral and cellular immune responses were characterized.

**Fig 3:**
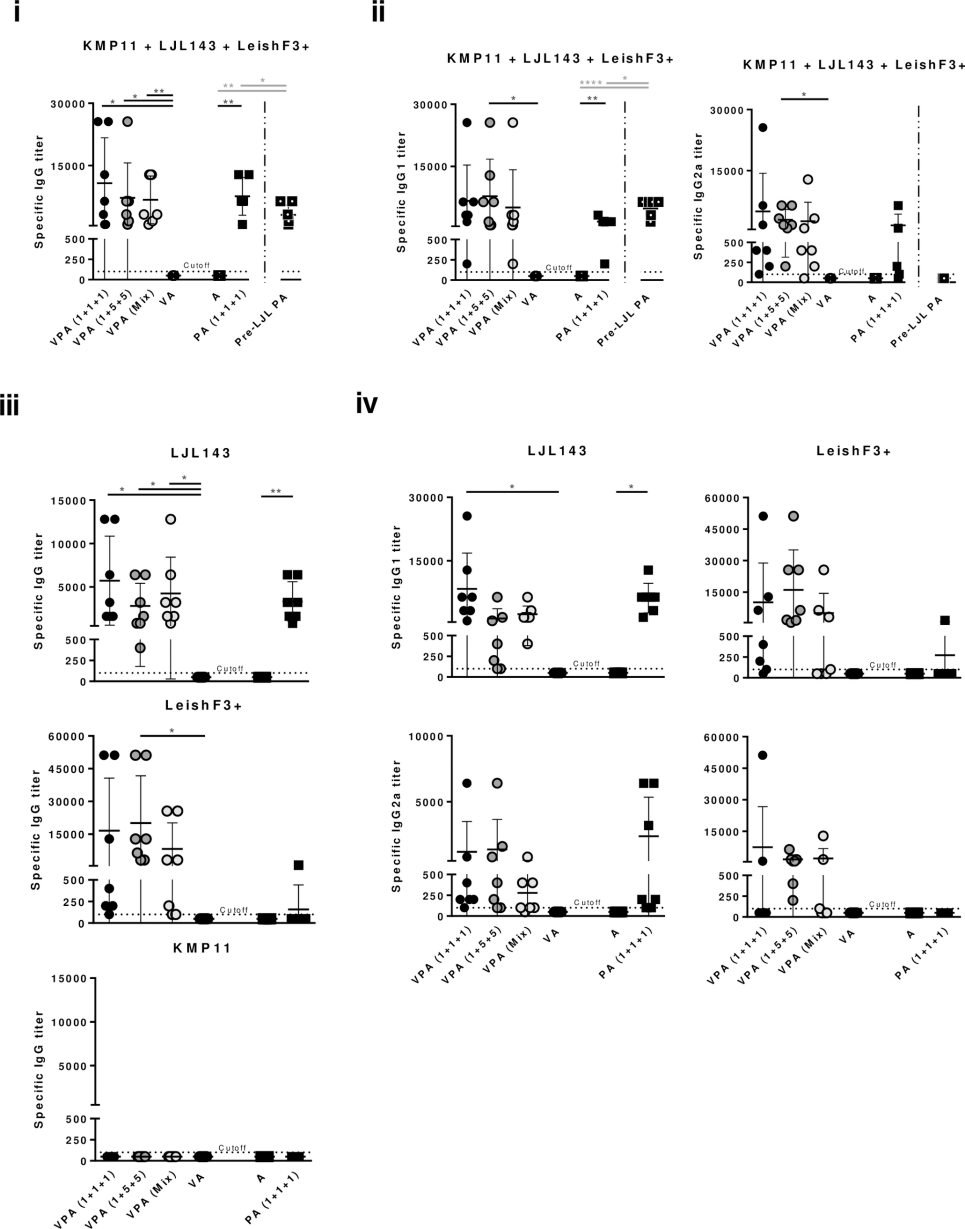
Antigen-specific vaccine-elicited humoral responses. Different experimental groups were designed for the development of the pre-clinical trials of the vaccine candidate in mice. Two groups represent negative controls: one composed by animals which received only the adjuvant (A), and other composed by animals that received the adjuvanted empty virosome (VA). The third group received non-formulated proteins with adjuvant in the dosage of 1 μg of each component [PA (1+1+1)]. The fourth was immunized with the same non-formulated proteins, but was primed with non-adjuvanted LJL143 three weeks before the first immunization (Pre-LJL PA). The three remaining groups received different formulations of adjuvanted formulated antigens (VPA). Two different VPA combinations were tested regarding antigen quantities: 1+1+1 or 1+5+5 indicate the administered dosages of formulated LJL143, KMP11 and LeishF3+ (individual virosome formulations). VPA (Mix) refers to the third virosome formulation tested, in which the three antigens (1μg each) were simultaneously formulated in the same virosome. Mice were immunized 3 times i.m. (separated by 4 weeks each), euthanized 4 weeks after the last immunization, and their sera collected. Specific IgG or IgG1 and IgG2a titers against the pool of antigens (i or ii, respectively), and against each individual antigen (iii or iv, respectively) were determined by ELISA. Each dot represents one animal. Average and SD of the values within each group are shown. Statistical differences are properly identified (One-Way ANOVA or Unpaired t-test (for comparison between primed and non-primed animals): * p≤0.05, ** p≤0.01 and **** p≤0.0001).

Antigen-specific humoral responses were determined in sera of vaccinated animals (normal immunization scheme) by ELISA. All groups showed specific responses against the pool of antigens, with comparable magnitudes among groups, and a mixed IgG1/IgG2a response (Fig 3i and 3ii). In contrast, the magnitude of antibody responses detected against individual antigens was distinct; the one against LeishF3+ was generally the highest and the one against KMP11 was non-detectable (Fig 3iii). Curiously, the virosome formulations promoted stronger humoral responses, specifically against LeishF3+ (Fig 3iii). To note, the best single-antigen specific isotype response was detected in the group of animals immunized with VPA (1+5+5) that showed a prevalence of IgG2a α-LJL143 and a mixed IgG1/IgG2a response against LeishF3+ (Fig 3iv).

**Fig 4:**
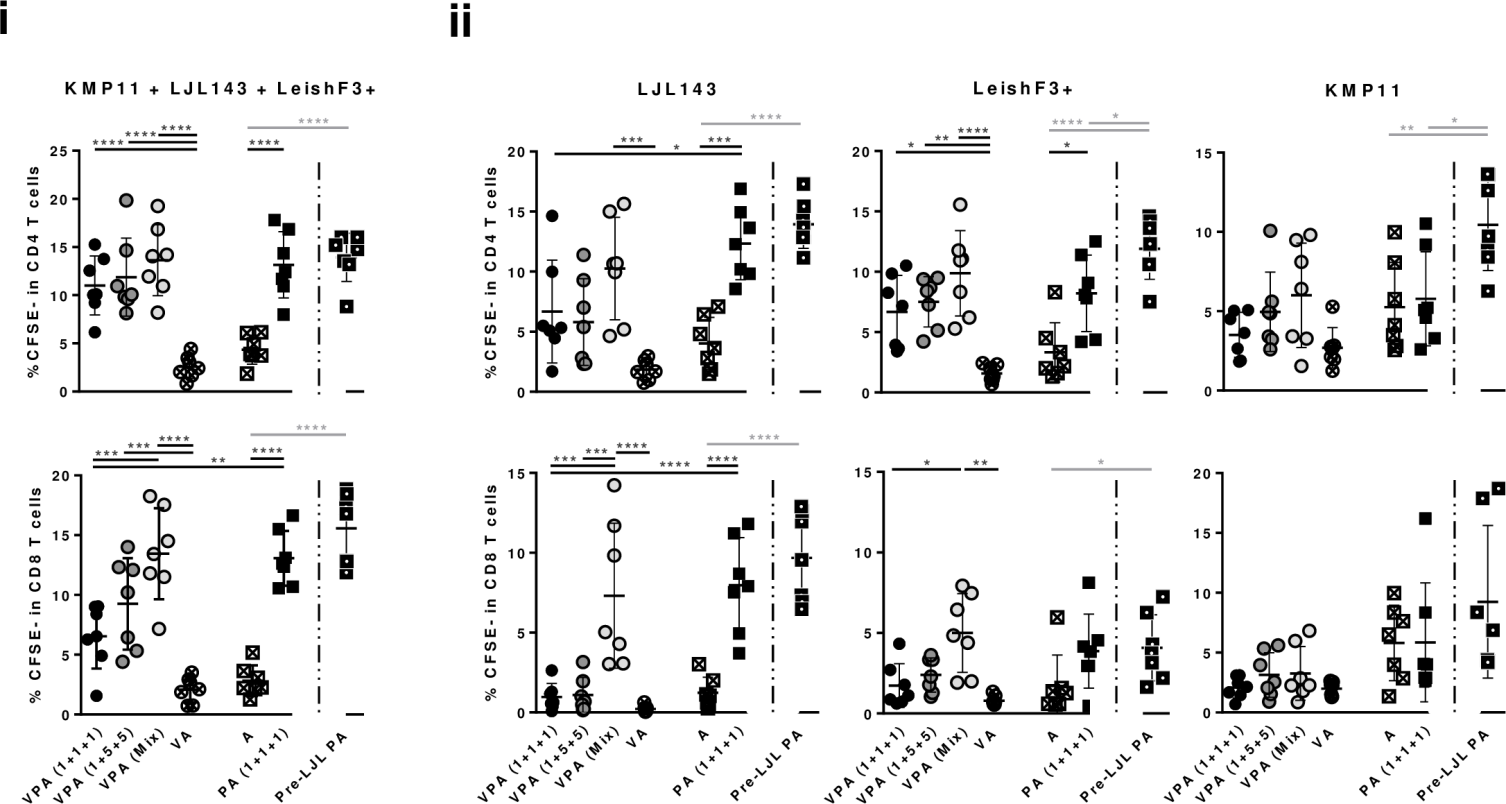
Antigen-specific vaccine-elicited T cell proliferation. Different experimental groups were designed for the development of the pre-clinical trials of the vaccine candidate in mice. Two groups represent negative controls: one composed by animals which received only the adjuvant (A), and other composed by animals that received the adjuvanted empty-virosome (VA). The third group received non-formulated proteins with adjuvant in the dosage of 1 μg of each component [PA (1+1+1)]. The fourth received the same non-formulated proteins, but was primed with non-adjuvanted LJL143 three weeks before the first immunization (Pre-LJL PA). The three remaining groups received different formulations of adjuvanted formulated antigens (VPA). Two different VPA combinations were tested regarding antigen quantities: 1+1+1 or 1+5+5 indicate the administered dosages of formulated LJL143, KMP11 and LeishF3+ (individual virosome formulations). VPA (Mix) refers to the third virosome formulation tested, in which the three antigens (1μg each) were simultaneously formulated in the same virosome. Mice were immunized 3 times i.m. (separated by 4 weeks each), euthanized 4 weeks after the last immunization, and their spleens collected and processed. Frequencies of proliferating splenic T cells (CD4^+^ and CD8^+^) were determined by flow cytometry, after four days of culture with the pool of antigens (10 μg/ml each) (i), or each of the individual antigens (10 μg/ml) (ii). Each dot represents one animal. Average and SD of the values within each group are shown. Statistical differences are properly identified (One-Way ANOVA or Unpaired t-test (for comparison between primed and non-primed animals): * p≤0.05, ** p≤0.01, *** p≤0.001 and **** p≤0.0001).

Antigen-specific cellular immune responses were determined through properly controlled cell proliferation assays using splenocytes. Overall, all the groups of vaccinated animals (normal immunization scheme) showed specific cell proliferation against the pool of antigens, at levels comparable among groups for proliferating CD4^+^ T cells (Fig 4i; p≤0.0001 *versus* controls), and with some minor differences among groups for CD8^+^ T cells (Fig 4i). Looking at the results of individual proliferation against each of the antigens, T cell responses to LJL143 were slightly stronger than those detected for LeishF3+, while no significant responses were detected against KMP11 (Fig 4ii). However, these differences in response-magnitude seem not to be due to an immunodominance of the sand fly-derived antigen, because results of the groups that received VPA (1+1+1) and VPA (1+5+5) are overall comparable (Fig 4ii). Interestingly, and in line with the observed response of human PBMCs, the responses obtained against LeishF3+ were higher in magnitude, than the ones previously obtained against LeishF3 (Fig 4ii *versus* S1i Fig; PA (1+1+1) *versus* PA). To note, both the CD4^+^ and CD8^+^ T cell specific responses were higher in the groups that received either PA (1+1+1) or VPA (Mix) (Fig 4i and 4ii).

**Fig 5:**
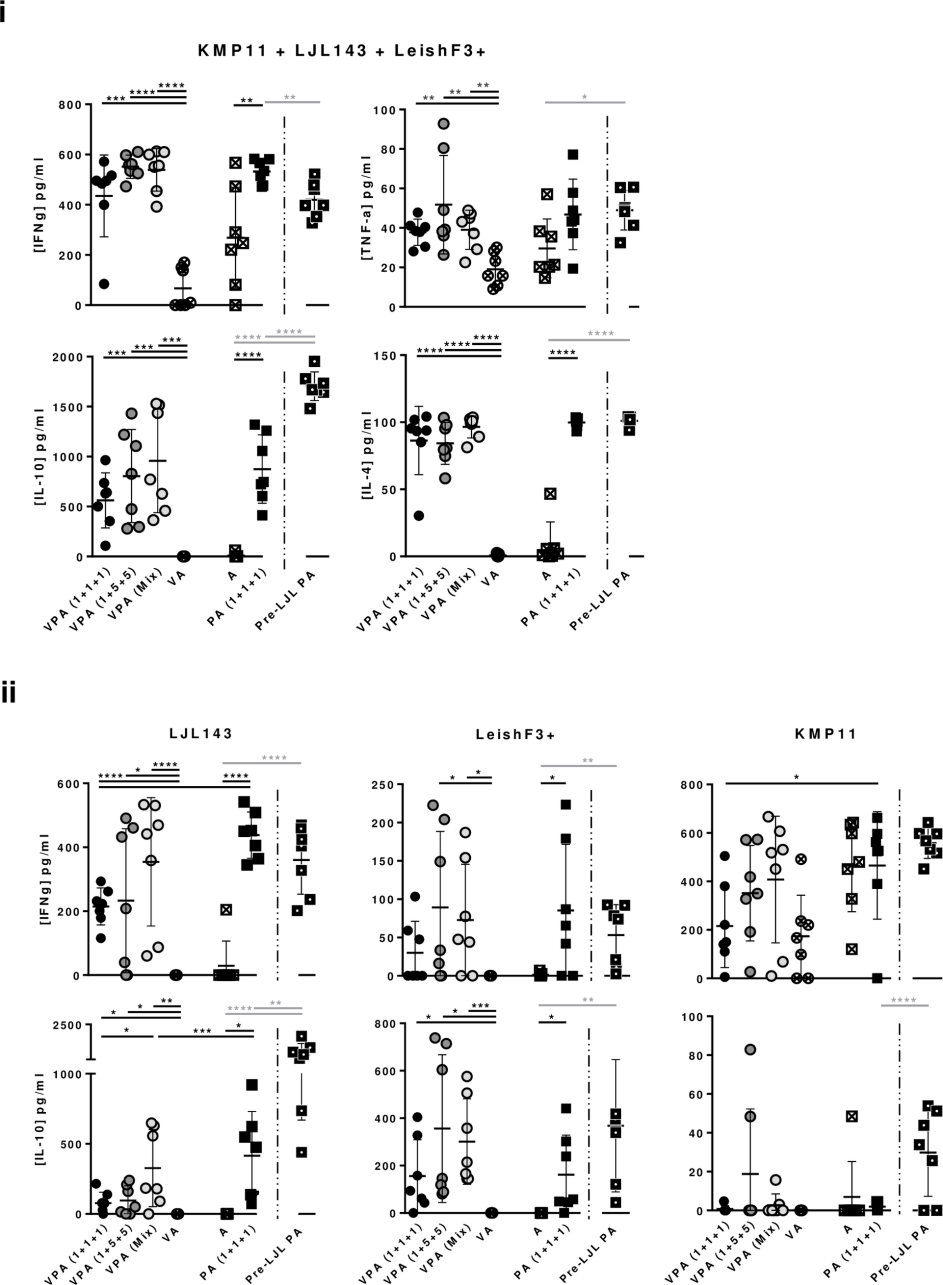
Antigen-specific vaccine-elicited cellular cytokine responses. Different experimental groups were designed for the development of the pre-clinical trials of the vaccine candidate in mice. Two groups represent negative controls: one composed by animals which received only the adjuvant (A), and other composed by animals that received the adjuvanted empty-virosome (VA). The third group received non-formulated proteins with adjuvant in the dosage of 1 μg of each component [PA (1+1+1)]. The fourth was immunized with the same non-formulated proteins, but was primed with non-adjuvanted LJL143 three weeks before the first immunization (Pre-LJL PA). The three remaining groups received different formulations of adjuvanted formulated antigens (VPA). Two different VPA combinations were tested regarding antigen quantities: 1+1+1 or 1+5+5 indicate the administered dosages of formulated LJL143, KMP11 and LeishF3+ (individual virosome formulations). VPA (Mix) refers to the third virosome formulation tested, in which the three antigens (1μg each) were simultaneously formulated in the same virosome. Mice were immunized 3 times i.m. (separated by 4 weeks each), euthanized 4 weeks after the last immunization, and their spleens collected. Typically pro-inflammatory and regulatory/anti-inflammatory cytokines were quantified by ELISA in the supernatants resultant from cellular proliferation assays against the pool of antigens (10 μg/ml each) (i). In parallel IFN-γ, and IL-10 levels were quantified by ELISA in the supernatants resultant from cellular proliferation assays against each of the individual antigens (10 μg/ml) (ii). Average and SD of the values within each group are shown. Statistical differences are properly identified (One-Way ANOVA or Unpaired t-test (for comparison between primed and non-primed animals): * p≤0.05, ** p≤0.01, *** p≤0.001 and **** p≤0.0001).

Cytokines quantified in the resulting supernatants from antigen-specific cell proliferation assays align with the cell proliferation results. All the groups of vaccinated animals (normal immunization scheme) showed specific production of both pro-inflammatory and regulatory/anti-inflammatory cytokines against the pool of antigens, at levels comparable among groups (Fig 5i; at least p≤0.01 comparing vaccinated groups with controls). The regulatory cytokine IL-10 was detected at higher levels, followed by IFN-γ, IL-4 and TNF-α, four days after splenocytes stimulation with the pool of antigens (Fig 5i). The cytokine response detected against each of the individual antigens was also a mirror of the cell proliferation results (Fig 5ii; S4 Fig). Of note, a comparable or relatively higher IFN-γ *versus* IL-10 response against LJL143 was detected in the groups that received PA (1+1+1) and VPA (MIX), or VPA (1+1+1) and VPA (1+5+5), respectively (Fig 5ii). LeishF3+ induces both IFN-γ and IL-10 responses in lower magnitudes, compared to the responses observed for LJL143, with the IFN-γ/IL-10 balance being higher in the group that received the non-formulated antigens (Fig 5ii). Additionally, and although unexpectedly high, the IFN-γ response detected against KMP11 was non-specific in nature (magnitude similar between vaccinated and control groups; Fig 5ii).

**Fig 6:**
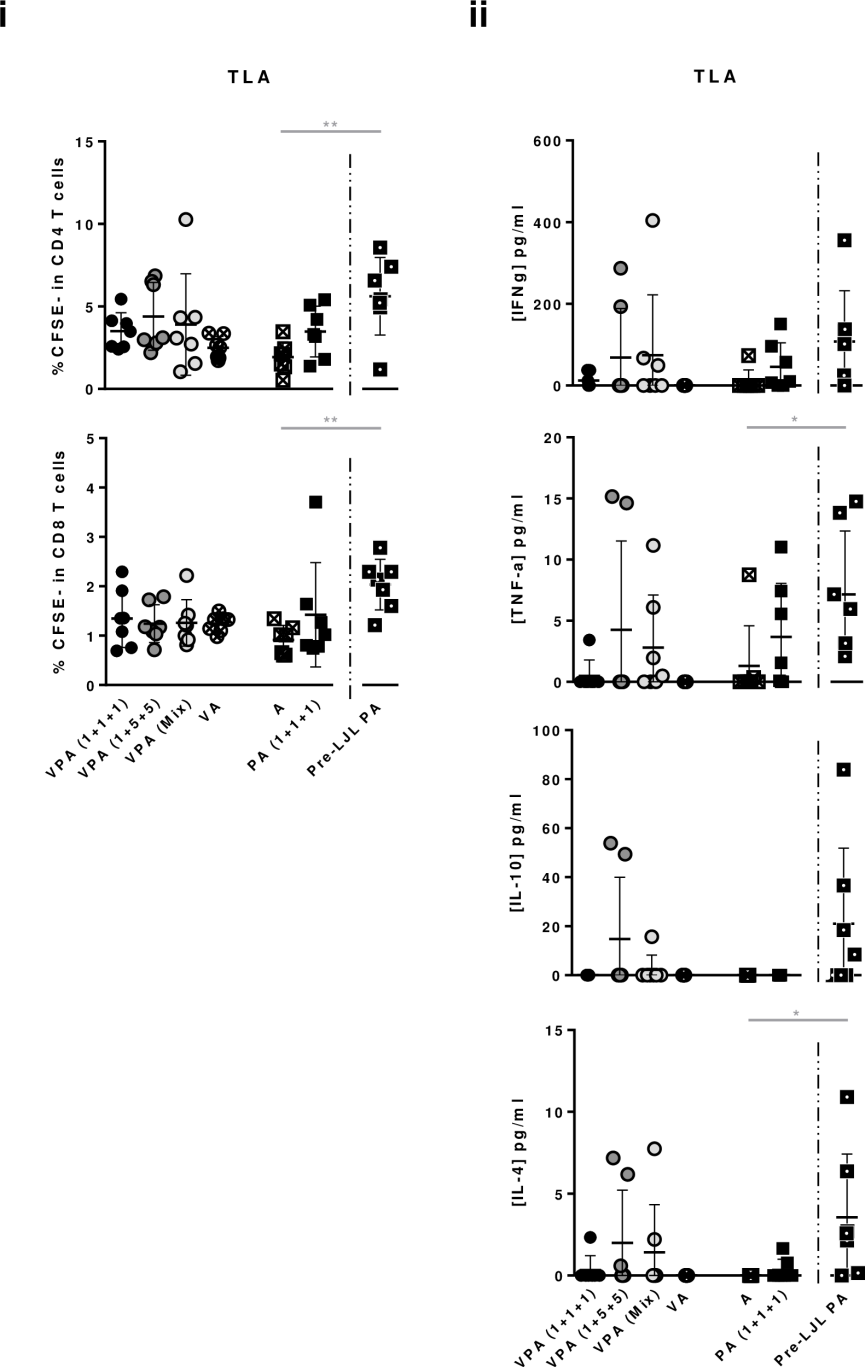
Determination of vaccine-elicited cellular immune responses against total parasite extract. Different experimental groups were designed for the development of the pre-clinical trials of the vaccine candidate in mice. Two groups represent negative controls: one composed by animals which received only the adjuvant (A), and other composed by animals that received the adjuvanted empty virosomes (VA). The third group received non-formulated proteins with adjuvant in the dosage of 1 μg of each component [PA (1+1+1)]. The fourth received the same non-formulated proteins, but was primed with non-adjuvanted LJL143 three weeks before the first immunization (Pre-LJL PA). The three remaining groups received different formulations of adjuvanted formulated antigens (VPA). Two different VPA combinations were tested regarding antigen quantities: 1+1+1 or 1+5+5 indicate the administered dosages of formulated LJL143, KMP11 and LeishF3+ (individual virosome formulations). VPA (Mix) refers to the third virosome formulation tested, in which the three antigens (1μg each) were simultaneously formulated in the same virosome. Mice were immunized 3 times i.m. (separated by 4 weeks each), euthanized 4 weeks after the last immunization, and their spleens collected. (i) Frequencies of splenic proliferating T cells were determined by flow cytometry, after four days of culture with Total *Leishmania* Antigen (TLA; equivalent to 10 parasites/cell). (ii) Cytokines were quantified by ELISA in the supernatants from the cellular proliferation assays. Average and SD of the values within each group are shown. Statistical differences are properly identified (One-Way ANOVA or Unpaired t-test (for comparison between primed and non-primed animals): * p≤0.05 and ** p≤0.01).

The cellular response of vaccinated mice against total parasite extract (Total *Leishmania* Antigens–TLA) was also evaluated through cell proliferation assays. While the proliferation levels of CD8^+^ T cells remained at the basal level, a tendency of increased CD4^+^ T cells proliferation was observed at comparable levels for all immunized groups, compared with controls (Fig 6i). Nevertheless, comparing the proliferation levels against TLA with the proliferation levels against the individual vaccine antigens, they were generally low (Fig 4ii *versus* Fig 6i). Cytokine production in response to TLA stimulation followed the same pattern: generally absent in the control groups *versus* variable in immunized groups (Fig 6ii; no statistically significant differences). A predominance of pro-inflammatory cytokines (mainly IFN-γ) were detected over regulatory/anti-inflammatory ones (Fig 6ii).

#### Priming with the sand fly salivary protein LJL143 enhances the response generated against parasite-derived antigens and produces a significant response to TLA

Previous observations [18, 19, 35, 36] support that a priming with a sand fly salivary protein alone and boosting with the sand fly protein together with other *Leishmania* antigens, may positively influence the outcome of posterior immunizations with complex vaccine formulations. To understand its potential, we tested within the antigenicity clinical trial, the effect of a previous administration of non-adjuvanted LJL143 on the response elicited by the non-formulated optimized vaccine.

In terms of the specific humoral immune response, priming with the salivary protein promoted an overall decrease in specific IgG titers against the pool of antigens with a prevalence of IgG1 isotype titers, in comparison with their counterparts (Fig 3i and 3ii; Pre-LJL PA versus PA (1+1+1); p≤0.05).

Interestingly, while the specific cellular response detected against the pool of proteins or the sand fly salivary protein were comparable between the primed and non-primed groups, and significantly different from the controls (Fig 4i; p≤0.0001), higher levels of proliferating CD4^+^ T cells against LeishF3+ and KMP11 or TLA were significantly, or tendentiously detected in the group of animals that was primed with the salivary protein LJL143 (Fig 4ii and Fig 6i; Pre-LJL PA *versus* PA (1+1+1); p≤0.05). Regarding CD8^+^ T cells, an apparent improvement of response was observed only against TLA when comparing primed with non-primed groups (Fig 6i). Cytokines quantified in the resulting supernatants from cell proliferation assays showed a robust production of IFN-γ against the pool of antigens in both primed and non-primed animals that was higher in the latter (Fig 5i; Pre-LJL PA *versus* PA (1+1+1); p≤0.01). In contrast, higher levels of IL-10 were detected against the pool of antigens in the group of animals that received the priming with LJL143 in comparison with its counterpart (Fig 5i; p≤0.0001). Comparing primed with non-primed groups, cytokine-secretion tendencies were maintained in response to LJL143, with a tendentiously lower production of IFN-γ and a significantly higher production of IL-10 (Fig 5ii; Pre-LJL PA *versus* PA (1+1+1); p≤0.01). A similar cytokine profile was detected in response to LeishF3+, while once more, the high IFN-γ levels detected in response to KMP11 were non-specific in origin (Fig 5ii). Remarkably, the cytokine response detected against TLA was higher in primed compared with non-primed groups [Fig 6ii; Pre-LJL PA *versus* PA (1+1+1)]. Of note, the production of IFN-γ in this group, in response to TLA, was five times higher in average than the secretion of IL-10 (Fig 6ii).

## Discussion

Because in *Leishmania* spp. endemic areas the majority of infected persons do not develop clinical symptoms and previous infection leads to robust immunity against the parasite, vaccination is considered as one of the most viable ways to control *Leishmania* infection. However, to date there is no anti-*Leishmania* vaccine available for humans [3, 8]. This work proposes an innovative vaccine concept, consisting on Virus-Like Particles (VLP) loaded with 3 different antigens, two from the parasite and one from the sand fly vector, adjuvanted with a TLR4 agonist, as a strong candidate to fill in the existing gap in terms of human anti-*Leishmania* vaccines.

Although already demonstrated as a useful adjuvant in the context of anti-*Leishmania* vaccination, we considered it essential to determine the effect of GLA-SE on vaccine-elicited immune responses, mainly because the vaccine candidate we propose is much more complex than the one previously tested (single recombinant fusion protein) [25], with a multi-antigen nature and a virosomal component, which may itself have an adjuvant effect [37]. As expected, the adjuvant generally improved the antigen-elicited immune response, both in terms of specific cellular and humoral responses elicited by non-formulated antigens (S1i and S1ii Fig; PA *versus* P). Furthermore, similar results were obtained for virosome-formulated proteins (Fig 1i and 1ii; VPA *versus* PA) indicating, on one hand, the essentiality of the adjuvant in this vaccination context, and on the other that the *Influenza* VLP are working mainly as vehicles, and not as adjuvants in this context.

For almost two decades in vaccinology, the effect of the antigen dosage in the final outcome of the immunization has been studied and discussed, always in parallel with the concept of antigen affinity [38, 39]. Here, in order to define the optimal vaccine composition, based on the specific responses elicited, we tested two doses of antigens and adjuvant. A lower antigen/adjuvant dose, although is worse regarding the humoral immune response elicited (Fig 1ii, S1ii Fig), promotes a stronger specific cellular immune response against both LJL143 and LeishF3 (Fig 1i, S1i Fig). These results point therefore to the idea that “less is more”, once it is generally accepted that cellular immunity is essential for *Leishmania* elimination [8], and justify the choice of the lower antigen/adjuvant dosages used in the pre-clinical trials *per se*.

In agreement with previous observations [40–42], we demonstrated the immunogenicity of KMP11 in humans. In fact, KMP11 was the antigen that generated a better response in the VL patients PBMCs stimulation experiments, with a significant increase in IFN-γ production by cells collected from cured VL patients compared with cells from matching endemic controls (Fig 2i). Such an observation was paramount to the final decision to include this particular antigen as a component of the innovative vaccine candidate. A possible explanation for the weak KMP11 immunogenicity detected in mice, which contrasts with previous studies in animals, is the use of the recombinant protein in opposition with the use of different DNA-based or heterologous recombinant live-vaccine approaches [21, 43–45].

On the other hand, the non-expressive response obtained in human *ex vivo* immunogenicity studies against LeishF3 (results generally similar between VL patients and controls; Fig 2i) was unexpected. A previous study showed the individual immunogenicity of NH and SMT, the two components of the fusion protein LeishF3, and successfully defined it as immunogenic and safe in a Phase I human clinical trial [25]. However, while the *ex vivo* immunogenicity assessment done by Coler and colleagues [25] was performed in a cohort from a *L*. *donovani* endemic area in Bangladesh, ours was performed using a cohort from a *L*. *infantum* endemic area in Spain, which may explain the lower-than-expected reactivity detected. These results led to the characterization of the immunogenicity of a LeishF3 “upgraded version” named LeishF3+ using the same human cohort, and the final substitution of LeishF3 by LeishF3+ in the vaccine formulation due to the observed improvement of the detected responses (Fig 2ii).

Interestingly, PBMCs from some individuals of the three different studied groups, including the controls (Old World human samples) responded to LJL143 (Fig 2i), a salivary protein from *Lutzomyia longipalpis*, the vector of VL in the New World. The sand fly salivary Lufaxin-like proteins are found in both the New and Old Worlds sand flies [46]. Within this family, LJL143 from *L*. *longipalpis* and PpeSP06, the homologous salivary protein from *P*. *perniciosus*, the main vector of VL in the Mediterranean Basin, share an amino acid sequence conservation of 45%. In line with this evidence, the reactivity, equally detected in samples from infected and non-infected individuals, is a potential indicator of immune cross-recognition of LJL143, with which in theory, the studied population has not been in contact before. These results further support the inclusion of this antigen in the vaccine formulation, stressing the sand fly salivary protein LJL143 as a potential “broad-spectrum antigen”.

All the above mentioned justifies the final composition of the optimized innovative vaccine used in the definitive pre-clinical trials in mice for extrapolation of its safety profile and characterization its in-depth immunogenic profile. Furthermore, several variables in the final outcome of the immunization, such as the contribution of the virosome to the induction of immunogenicity, or the possibility of immunodominance of the sand fly salivary protein, were considered.

The values of hematological studies, splenic cell populations, CD4^+^ T cell non-specific reactivity and IgE specific titers (Table 1, S3i–S3iv Fig), determined in the pre-clinical trials, potentially indicate the safety of each vaccine component (proteins, adjuvant and virosome). The absence of specific IgE titers deserves to be highlighted, due to the correlation of antigen-specific IgE and vaccine-associated anaphylatic reactions development, shown particularly, but not exclusively for anti-*Influenza* vaccines [47]. To further explore the safety of the vaccine candidate, a repeated dose toxicity study in rabbits, complying with the WHO Expert Committee on Biological Standardization [48] is ongoing.

The different optimized formulations tested are indeed immunogenic, eliciting overall significant specific humoral and cellular immune responses (Figs 3i–3iv and 4i and 4ii). Regarding the humoral responses detected, they were generally mixed in nature (IgG1/IgG2a), indicating a mixed Th1/Th2 phenotype. Furthermore, the improvement of the specific humoral response against LeishF3+ induced by the VLP-based antigen formulations (Fig 3iii) deserves to be highlighted, as a possible advantage of the use of formulated antigens without forgetting, however, the debatable relevance of the humoral immune responses in the context of VL [49]. In respect to cellular immune responses detected, they shown distinct magnitudes, depending on each of the individual antigens. Reproducibly, the sand fly-derived antigen induced a more robust response than the parasite-derived ones (LJL143 ≥ LeishF3+ > KMP11; Fig 4ii). Of note, the responses obtained against LeishF3+ were higher than those previously obtained against LeishF3 (Fig 4ii *versus* S1i Fig; PA (1+1+1) *versus* PA). This difference in the magnitude of the responses detected against the three different antigens seems not to be an immunodominance problem, since both cellular and humoral immune responses detected against LeishF3+ (the parasite derived antigen showing significant responses) were similar for the groups that received VPA (1+1+1) and VPA (1+5+5) (doses of LJL143, KMP11 and LeishF3+, respectively; Figs 3iii and 4ii).

Although it is a dogma that the protection against *Leishmania spp*. requires antigen-specific CD4^+^ and CD8^+^ T cell responses, the correlates of immunity to human VL are yet to be completely understood [50]. Therefore, the vaccine correlates of protection are still a debatable issue that takes bigger proportions when we add the translatability of animal pre-clinical trials to the equation. This said, the balance between specific IFN-γ and IL-10 production, has been used as predictive of vaccine efficacy in mice [51, 52]. In our study, through cell proliferation assays we detected in non-primed groups, a higher production of IL-10 than IFN-γ in response to the pool of antigens or to LeishF3+ alone, an either comparable or prevalent IFN-γ over IL-10 response against LJL143 (depending on the experimental group; Fig 5i and 5ii) and a limited but prevalent IFN-γ over IL-10 response against total parasite antigens (TLA; Fig 6ii). The Th1 directed response induced by stimulation with TLA, the experimentally closest experimental set up to the infectious process (deposition of whole parasites in the skin) is a promising indication of vaccine effectiveness. Nevertheless, we cannot ignore the apparent main Th2 response induced by the pool of antigens and LeishF3+, and either mixed or Th1 responses induced by LJL143. One curious observation is that, while the IL-10 levels quantified in the cell proliferation against the pool of antigens are 1.5 fold higher than the sum of levels determined in the cell proliferation against the individual antigens (excluding KMP11), the same comparison gives similar IFN-γ (Fig 5i and 5ii). This particular observation, together with evidence showing that stimulation with high antigen doses leads to enhanced IL-10 production by Th1 CD4^+^ cells [53], makes us speculate on the occurrence of a possible regulatory mechanism *in vitro* as a way to control a vigorous immune response and prevent inflammation-mediated damage.

In parallel, in the pre-clinical trial, we evaluated the effect of priming with the sand fly salivary protein in the final vaccine-elicited immune responses. Interestingly, the previous administration of the sand fly saliva derived antigen may be beneficial for the generation of a better response against the parasite-derived antigens, particularly in terms of cellular immunity (higher CD4^+^ T cell proliferation against LeishF3+, KMP11 and TLA; Figs 4ii and 6i). Nevertheless, the IL-10 response detected, particularly against the pool of antigens and LJL143, was higher in the primed animals (Fig 5i and 5ii). On the other hand, the specific IFN-γ response generated by TLA increased tendentiously comparing primed with non-primed animals, and was 5 fold higher than the TLA induced IL-10 response (Fig 6ii), making this vaccination approach interesting to be tested in terms of anti-*Leishmania* effectiveness, in the context of natural infection (parasites delivered by the sand fly in the presence of salivary proteins).

Overall our results indicate that the innovative vaccine candidate tested here represents a promising anti-*Leishmania* vaccine. Some questions remain that need to be further explored, such as the potential benefits or implications of the predicted constant vector exposure in endemic countries, as well as a probable exposure to *Influenza* virus, to the final vaccine-induced responses.

## Supporting information

S1 Fig Effect of antigen/adjuvant doses in the response generated by non-formulated proteins. Groups of BALB/c mice were immunized 3 times i.m. (separated by 4 weeks each) with 2 different doses of adjuvant/antigens: 1 μg and 5 μg of each individual component (PA). Non-adjuvanted antigens (P; 1 μg of each antigen) or PBS were injected in controls. Four weeks after the last immunization, animals were euthanized and their spleens and sera collected. Specific CD4^+^ and CD8^+^ (i) T cell proliferation was assessed by Flow Cytometry four days after CFSE-stained splenocytes culture in the presence of each of the single non-formulated antigens (10 μg/ml). (ii) Serum antigen specific IgG titers for each of the antigens were determined by ELISA. Each dot represents one animal. Average and SD of the values within each group are shown. Statistical differences are properly identified (Unpaired t-test: * p≤0.05, ** p≤0.01, *** p≤0.001 and **** p≤0.0001).
(TIF)

S2 Fig Representation of the pre-clinical trial *per-se* timeline. M represents month. W represents week. V represents virosome. A represents adjuvant. P represents proteins or antigens. Numbers 1+1+1 or 1+5+5 in brackets represent the administered doses in μg of LJL143, LeishF3+ and KMP-11, respectively. VPA(Mix) represents one formulation in which the three antigens (1μg each) were simultaneously formulated in the same virosome, contrarily to the other two VPA formulations that are mixtures of individual virosomal antigen preparations.
(TIF)

S3 Fig Extrapolation of the safety profile of the vaccine components. Different experimental groups were designed for the development of the pre-clinical trials of the vaccine candidate in mice. Two groups represent negative controls: one composed by animals which received only the adjuvant (A), and other composed by animals that received the adjuvanted empty-virosome (VA). The third group received non-formulated proteins with adjuvant in the dosage of 1 μg of each component [PA (1+1+1)]. The fourth received the same non-formulated proteins, but was primed with non-adjuvanted LJL143 three weeks before the first immunization (Pre-LJL PA). The three remaining groups received different formulations of adjuvanted formulated antigens (VPA). Two different VPA combinations were tested regarding antigen quantities: 1+1+1 or 1+5+5 indicate the administered dosages of formulated LJL143, KMP11 and LeishF3+ (individual virosome formulations). VPA (Mix) refers to the third virosome formulation tested, in which the three antigens (1μg each) were simultaneously formulated in the same virosome. Mice were immunized 3 times i.m. (separated by 4 weeks each), euthanized 4 weeks after the last immunization, and their spleens and sera collected. (i) Spleen weights and total cell numbers were determined. Myeloid (ii) and lymphoid (iii) splenic cell populations frequencies were determined by Flow Cytometry, and translated to absolute numbers. (iv) Antigen-specific IgE titers were determined by ELISA (individually against LJL143, LeishF3+ and KMP-11). Average and SD of the values within each group are shown. Statistical differences are properly identified (One-Way ANOVA: * p≤0.05, ** p≤0.01 and *** p≤0.001).
(TIF)

S4 Fig Specific vaccine-elicited cellular cytokine responses. Different experimental groups were designed for the development of the pre-clinical trials of the vaccine candidate in mice. Two groups represent negative controls: one composed by animals which received only the adjuvant (A), and other composed by animals that received the adjuvanted empty virosome (VA). The third group received non-formulated proteins with adjuvant in the dosage of 1 μg of each component [PA (1+1+1)]. The fourth received the same non-formulated proteins, but was primed with non-adjuvanted LJL143 three weeks before the first immunization (Pre-LJL PA). The three remaining groups received different formulations of adjuvanted formulated antigens (VPA). Two different VPA combinations were tested regarding antigen quantities: 1+1+1 or 1+5+5 indicate the administered dosages of formulated LJL143, KMP11 and LeishF3+ (individual virosome formulations). VPA (Mix) refers to the third virosome formulation tested, in which the three antigens (1μg each) were simultaneously formulated in the same virosome. Mice were immunized 3 times i.m. (separated by 4 weeks each), euthanized 4 weeks after the last immunization, and their spleens collected. TNF-α, and IL-4 levles were quantified by ELISA in the supernatants resultant from cellular proliferation assays against each of the individual antigens. Data presented refers only to non-primed animals. Each dot represents one animal. Average and SD of the values within each group are shown. Statistical differences are properly identified (One-Way ANOVA: * p≤0.05, ** p≤0.01, *** p≤0.001 and **** p≤0.0001).
(TIF)
