# Supplementary figures and images for: Pre-clinical antigenicity studies of an innovative multivalent vaccine for human visceral leishmaniasis
Source: PLoS Negl Trop Dis. 2017 Nov 27;11(11):e0005951. doi: 10.1371/journal.pntd.0005951 (PMC5720812; doi:10.1371/journal.pntd.0005951)

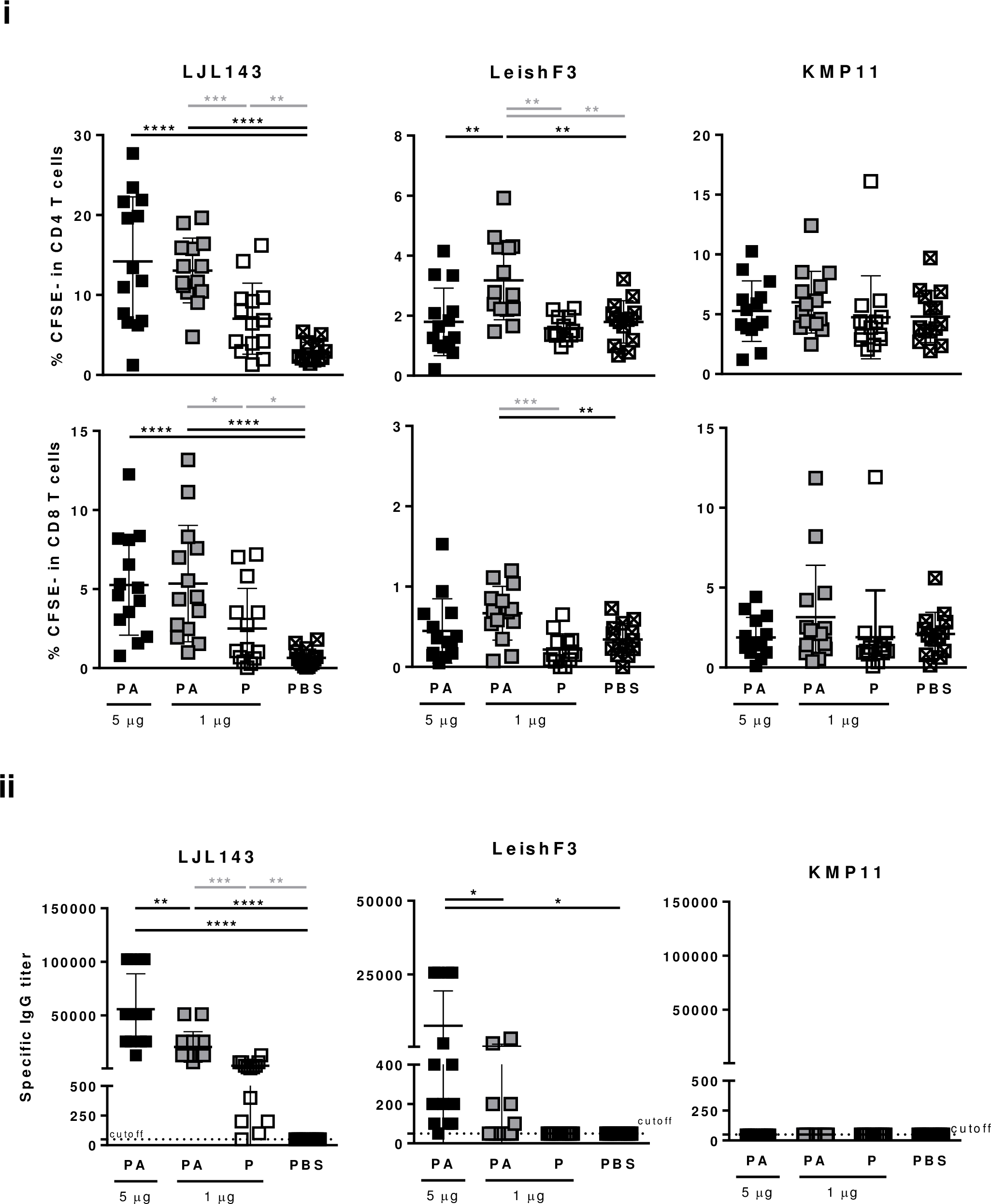

Supplement: S1 Fig — Groups of BALB/c mice were immunized 3 times i.m. (separated by 4 weeks each) with 2 different doses of adjuvant/antigens: 1 μg and 5 μg of each individual component (PA). Non-adjuvanted antigens (P; 1 μg of each antigen) or PBS were injected in controls. Four weeks after the last immunization, animals were euthanized and their spleens and sera collected. Specific CD4+ and CD8+ (i) T cell proliferation was assessed by Flow Cytometry four days after CFSE-stained splenocytes culture in the presence of each of the single non-formulated antigens (10 μg/ml). (ii) Serum antigen specific IgG titers for each of the antigens were determined by ELISA. Each dot represents one animal. Average and SD of the values within each group are shown. Statistical differences are properly identified (Unpaired t-test: * p≤0.05, ** p≤0.01, *** p≤0.001 and **** p≤0.0001). (TIF) [file pntd.0005951.s001.tif]

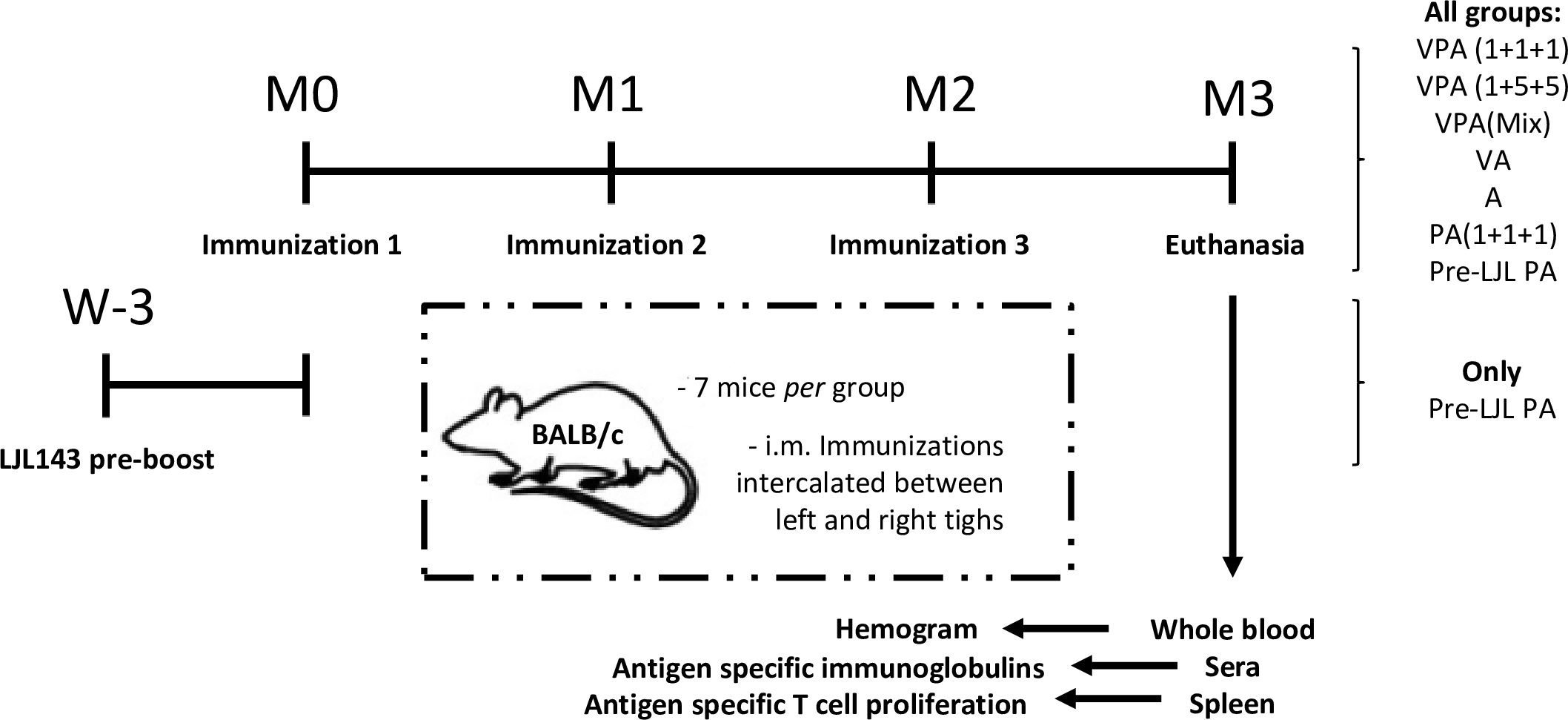

Supplement: S2 Fig — M represents month. W represents week. V represents virosome. A represents adjuvant. P represents proteins or antigens. Numbers 1+1+1 or 1+5+5 in brackets represent the administered doses in μg of LJL143, LeishF3+ and KMP-11, respectively. VPA(Mix) represents one formulation in which the three antigens (1μg each) were simultaneously formulated in the same virosome, contrarily to the other two VPA formulations that are mixtures of individual virosomal antigen preparations. (TIF) [file pntd.0005951.s002.tif]

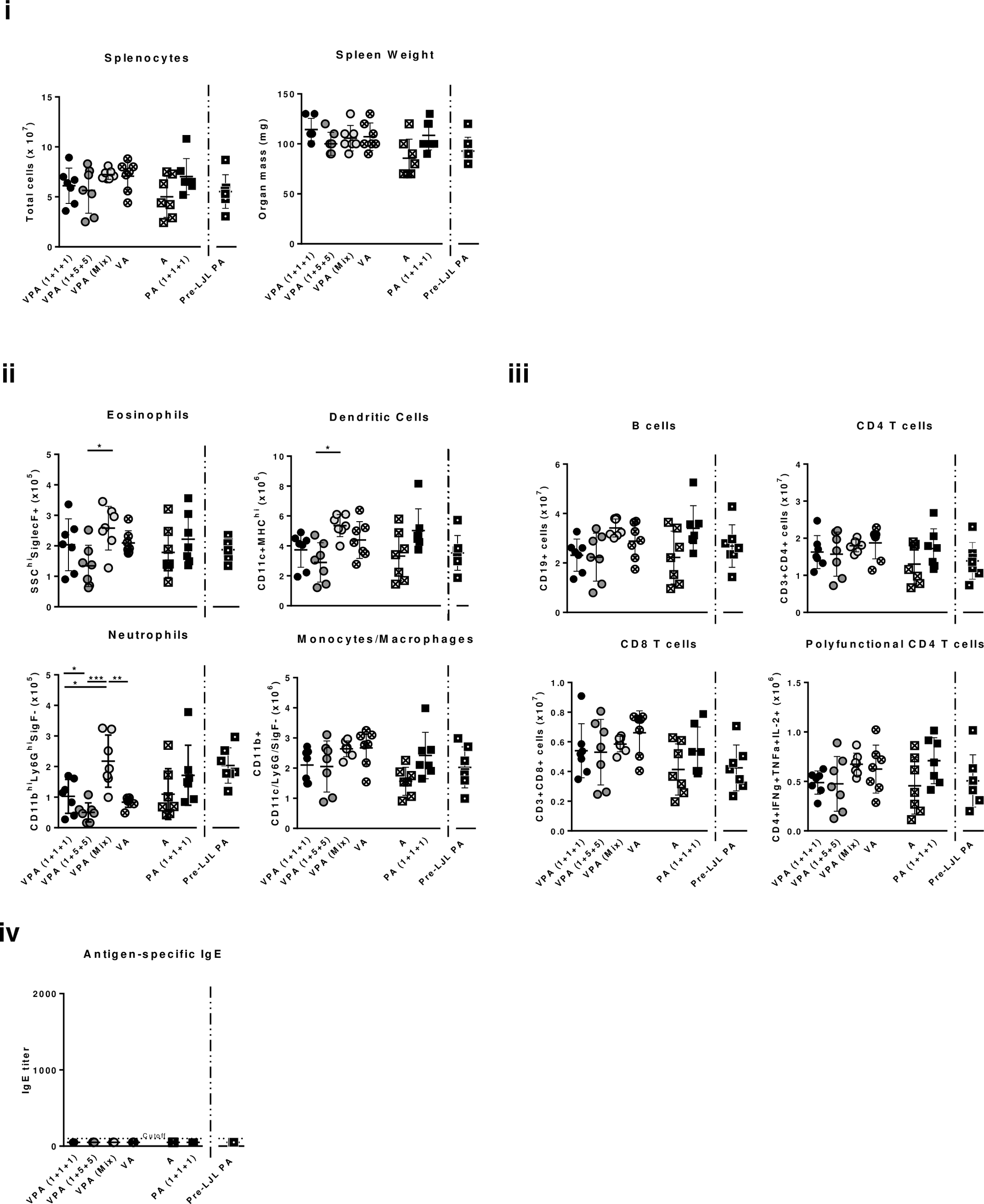

Supplement: S3 Fig — Different experimental groups were designed for the development of the pre-clinical trials of the vaccine candidate in mice. Two groups represent negative controls: one composed by animals which received only the adjuvant (A), and other composed by animals that received the adjuvanted empty-virosome (VA). The third group received non-formulated proteins with adjuvant in the dosage of 1 μg of each component [PA (1+1+1)]. The fourth received the same non-formulated proteins, but was primed with non-adjuvanted LJL143 three weeks before the first immunization (Pre-LJL PA). The three remaining groups received different formulations of adjuvanted formulated antigens (VPA). Two different VPA combinations were tested regarding antigen quantities: 1+1+1 or 1+5+5 indicate the administered dosages of formulated LJL143, KMP11 and LeishF3+ (individual virosome formulations). VPA (Mix) refers to the third virosome formulation tested, in which the three antigens (1μg each) were simultaneously formulated in the same virosome. Mice were immunized 3 times i.m. (separated by 4 weeks each), euthanized 4 weeks after the last immunization, and their spleens and sera collected. (i) Spleen weights and total cell numbers were determined. Myeloid (ii) and lymphoid (iii) splenic cell populations frequencies were determined by Flow Cytometry, and translated to absolute numbers. (iv) Antigen-specific IgE titers were determined by ELISA (individually against LJL143, LeishF3+ and KMP-11). Average and SD of the values within each group are shown. Statistical differences are properly identified (One-Way ANOVA: * p≤0.05, ** p≤0.01 and *** p≤0.001). (TIF) [file pntd.0005951.s003.tif]

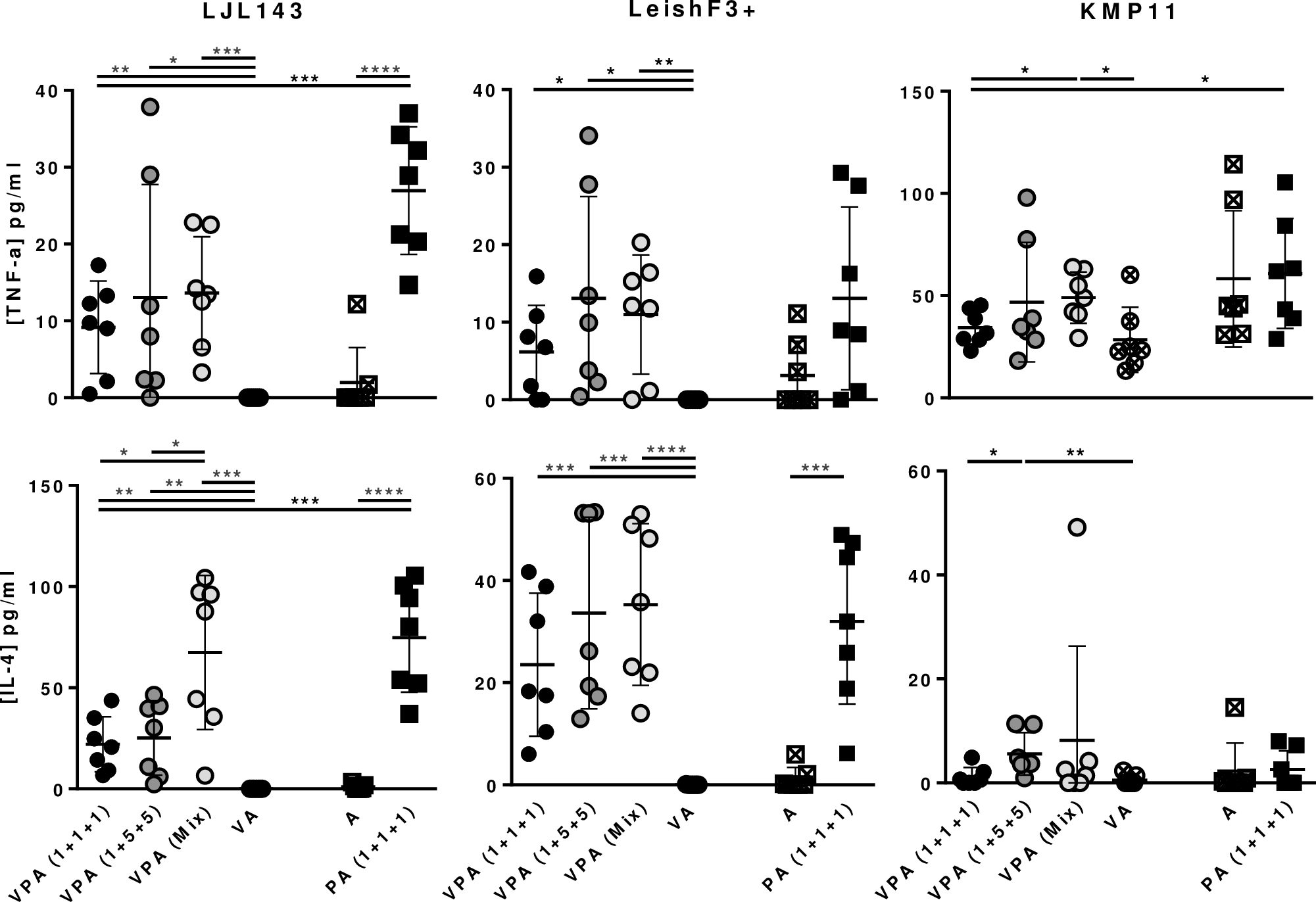

Supplement: S4 Fig — Different experimental groups were designed for the development of the pre-clinical trials of the vaccine candidate in mice. Two groups represent negative controls: one composed by animals which received only the adjuvant (A), and other composed by animals that received the adjuvanted empty virosome (VA). The third group received non-formulated proteins with adjuvant in the dosage of 1 μg of each component [PA (1+1+1)]. The fourth received the same non-formulated proteins, but was primed with non-adjuvanted LJL143 three weeks before the first immunization (Pre-LJL PA). The three remaining groups received different formulations of adjuvanted formulated antigens (VPA). Two different VPA combinations were tested regarding antigen quantities: 1+1+1 or 1+5+5 indicate the administered dosages of formulated LJL143, KMP11 and LeishF3+ (individual virosome formulations). VPA (Mix) refers to the third virosome formulation tested, in which the three antigens (1μg each) were simultaneously formulated in the same virosome. Mice were immunized 3 times i.m. (separated by 4 weeks each), euthanized 4 weeks after the last immunization, and their spleens collected. TNF-α, and IL-4 levles were quantified by ELISA in the supernatants resultant from cellular proliferation assays against each of the individual antigens. Data presented refers only to non-primed animals. Each dot represents one animal. Average and SD of the values within each group are shown. Statistical differences are properly identified (One-Way ANOVA: * p≤0.05, ** p≤0.01, *** p≤0.001 and **** p≤0.0001). (TIF) [file pntd.0005951.s004.tif]
